# Response of Fibroblasts MRC-5 to Flufenamic Acid-Grafted MCM-41 Nanoparticles

**DOI:** 10.3390/bioengineering5010004

**Published:** 2018-01-09

**Authors:** Giovanna Gomes Lara, Marcelo Fernandes Cipreste, Gracielle Ferreira Andrade, Wellington Marcos da Silva, Edésia Martins Barros de Sousa

**Affiliations:** Centro de Desenvolvimento da Tecnologia Nuclear—CDTN—Avenida Presidente Antônio Carlos, 6.627-Campus UFMG, Belo Horizonte CEP 31270-901, Minas Gerais, Brazil; gilara2009@gmail.com (G.G.L.); mcipreste@gmail.com (M.F.C.); graciellefandrade@yahoo.com (G.F.A.); wellingtonmarcos@yahoo.com.br (W.M.d.S.)

**Keywords:** MCM-41, flufenamic acid, MRC-5 cells, functionalization, biocompatibility

## Abstract

Recently, flufenamic acid (FFA) was discovered among fenamates as a free radical scavenger and gap junction blocker; however, its effects have only been studied in cancer cells. Normal cells in the surroundings of a tumor also respond to radiation, although they are not hit by it directly. This phenomenon is known as the bystander effect, where response molecules pass from tumor cells to normal ones, through communication channels called gap junctions. The use of the enhanced permeability and retention effect, through which drug-loaded nanoparticles smaller than 200 nm may accumulate around a tumor, can prevent the local side effect upon controlled release of the drug. The present work, aimed at functionalizing MCM-41 (Mobil Composition of Matter No. 41) silica nanoparticles with FFA and determining its biocompatibility with human fibroblasts MRC-5 (Medical Research Council cell strain 5). MCM-41, was synthesized and characterized structurally and chemically, with multiple techniques. The biocompatibility assay was performed by Live/Dead technique, with calcein and propidium–iodide. MRC-5 cells were treated with FFA-grafted MCM-41 for 48 h, and 98% of cells remained viable, without signs of necrosis or morphological changes. The results show the feasibility of MCM-41 functionalization with FFA, and its potential protection of normal cells, in comparison to the role of FFA in cancerous ones.

## 1. Introduction

The International Agency for Cancer Research (IACR) and Globocan registered that approximately 17,100,000 new cases of cancer are expected worldwide by 2020. Besides surgery and chemotherapy, radiotherapy is often used in cancer patients to eliminate or control the growth of tumors. The radiation doses can reach up to 50 Gy [1,2,3]. Technological advances have led to an improvement in radiation therapy, by which smaller areas of normal tissue are hit by ionizing radiation. However, the normal cells in the surroundings of the tumor are more prone to toxic effects from signaling molecules that can genomically destabilize those cells. Patients with breast cancer subjected to a fractioned dose of 39 Gy showed dermatitis in 24% of cases, while dermatitis increased to 36% in those who received 50 Gy [4]. 

Recently, flufenamic acid (FFA) was discovered among fenamates as a free radical scavenger and gap junction blocker [5]. Inside the cells, H_2_O_2_ is reduced by an enzyme known as glutathione reductase (GSH). In absence of oxidative stabilizing agents, such as divalent cations (e.g., Ca^2+^, Mg^2+^), the GSH tends to accumulate in the extracellular space, causing apoptosis of astrocytes. This effect was lowered after treating the cells with FFA at 100 µM, which decreased the GSH accumulation to approximately 65% [6]. FFA, as well as other non-steroid anti-inflammatory drugs (NSAIDs), induces apoptosis of cancer cells and have been reported to lower the risks of colorectal adenomas [7,8]. 

The use of the enhanced permeability and retention (EPR) effect, through which drug-loaded nanoparticles smaller than 200 nm may accumulate around the tumor, can prevent the local side effect upon controlled release of the drug [9].

Mesoporous silica nanoparticles can carry weakly water-soluble drugs, and protect most of them from degradation by extracellular enzymes [10]. Some features of mesoporous silica nanoparticles make them attractive for use in diagnostics and treatment of cancer. Its high specific surface area, controllable pore diameter, and volume, as well as the pore size distribution, allow for the tailoring of its features according to the drug to be grafted in the nanoparticle [11,12]. Ellahioui and colleagues reviewed reports on the cytotoxicity of silica nanoparticles, and its applications in the biomedical field, as carriers of anti-inflammatory drugs [13]. Considering this, it is possible to anchor many kinds of molecules on the silica mesoporous surface, such as directing agents, as well as for rendering the nanoparticles less cytotoxic by controlling their surface charges. 

Nanostructured silica nanoparticles are hosts of a specific drug, which is released depending on certain parameters, such as pore size, pore shape, and the interaction between the drug and the mesoporous solid [14]. Many non-steroidal anti-inflammatory drugs have been loaded to silica nanoparticles, such as diclofenac, to test their release in an acid stomach pH environment. It was found that the silica nanoparticle was able to retain diclofenac for a longer period of time in acidic pH [15]. Another research group reported the aminated surface of MCM-41 as an anchoring site for budesonide. The budesonide-grafted MCM-41 protected HT-29 (human colon adenocarcinoma cell line) cells from further damage in an inflammatory bowel disease model [16]. To get the best control in the release of the drugs, and to obtain a greater adsorption of the drugs, modifications of the chemical surface on the host matrices are necessary and crucial. The main alternative for those modifications is the post-synthesis functionalization by subsequent surface modification, or grafting, which provides good preservation of the mesostructure after treatment, as well as advantageous surface properties, such as a higher selectivity of a specific adsorbent, as proved by Sousa et al. [17].

Functionalization of aminated MCM-41 with folic acid (FA) has been successful after activation of a carboxyl group with 1-Ethyl-3-(3-dimethylaminopropyl)carbodiimide (EDC) and N-hydroxysuccinamide (NHS) [17,18]. In addition, treatment of BALB/c mice with a previously aminated MCM-41, followed by grafting with FA, showed that silica nanoparticles were able to reach and accumulate in the tumor, as determined by ex vivo fluorescence imaging [19]. In addition, silica nanoparticles alone showed biocompatibility above 60% with most cell lines tested [20,21,22,23]. Moreover, the nanoparticles elicit low immune response, determined by low production of the most common inflammatory cytokines, known as interleukin-6, interleukin-12, and 1β in vivo [24]. Another experiment done in vivo, in nude mice with labelled silica nanoparticles and assessed by ultrasound, showed low cytotoxicity; in three days, the silica started to degrade from inside out in an in vitro test [25]. Functionalization of silica nanoparticles with 3-Aminopropyltriethoxysilane (APTES) for surface amination, using the silanol groups as anchoring sites, have been reported by various groups, followed by further grafting with anti-cancer drugs or tumor-directing agents [26,27]. Although some drugs are already available in nanocarriers, such as liposomes and silica, FFA is not available in any type of nanoparticle employed for clinical use. 

Taking all these considerations into account, and due to the lack of reports on the effects of FFA in normal cells, the present work is aimed primarily at the functionalization of MCM-41 with FFA, the characterization of this system, and determining its biocompatibility to MRC-5 fibroblasts, in order to verify the potential of FFA grafted onto silica MCM-41 surfaces to protect normal cells when exposed to radiation.

## 2. Materials and Methods

### 2.1. Materials

The materials used for synthesis, functionalization, cell culture, and cell staining processes, and their respective manufacturers, are as follows: cetyltrimethylammonium bromide (CTAB), Tetraethylorthosilicate (TEOS), 3-Aminopropyltriethoxysilane (APTES), Dimethylaminopropyl)-N’-ethylcarbodiimide (EDC), N-Hydroxysuccinimide (NHS), flufenamic acid (FFA), Dulbecco’s Modified Eagles’s Medium- high glucose (DMEM), penicillin, streptomycin andamphotericin (PSA), and fetal bovine serum, which were all purchased from Sigma-Aldrich. Calcein-Orange and Hoescht 33258 were purchased from Invitrogen, ethanol from EMSURE, toluene from FMAIA, and Dimethyl Sulfoxide (DMSO) from ACRÓS ORGANICS. 

### 2.2. Synthesis of MCM-41

MCM-41 silica was prepared in accordance to a published procedure [17], using commercial CTAB as a template agent in basic conditions. CTAB (2.74 mmol) and NaOH (7.00 mmol) were dissolved in 480 mL of Milli-Q^®^ water. The temperature of the mixture was adjusted to 78 °C. TEOS (22.4 mmol) was added dropwise to the surfactant solution under vigorous stirring. The mixture was allowed to react for 2 h. After reaction, the mixture was filtered, washed with water and methanol, and dried at 60 °C for 24 h. The surfactant was removed by calcination, which was carried out by increasing the temperature to 550 °C under nitrogen flow for 2 h, followed by 3 h in air. 

### 2.3. Functionalization of MCM-41 with APTES: MCM-41/AP 

The first step in the functionalization process was to aminate the surface of MCM-41 to receive FFA moieties. Briefly, 27.5 mL of toluene was poured in a 50 mL round bottle. Then, 400 mg of MCM-41 was dispersed in the bottle, under constant stirring. Next, 156 µL of APTES was added, and this mixture was kept under constant magnetic stirring at 80 °C for 24 h. The suspension was filtered and washed thoroughly with Milli-Q^®^ H_2_O and ethanol (70%), followed by drying at 37 °C for two days. This sample was named MCM-41/AP. 

### 2.4. Functionalization of MCM-41/AP with FFA: MCM-41/AP-FFA

In the next step, 22.5 mL of DMSO was poured in a 50 mL round bottle, then 60 mg of EDC, 60 mg of NHS, and 162 mg of FFA was added under magnetic stirring. After 1 h, 200 mg of MCM-41/AP was added, and the reaction occurred overnight at room temperature. The suspension was filtered, washed, and dried as mentioned previously, and the sample was named MCM-41/AP-FFA, following proportions from the standard protocol.

### 2.5. Physicochemical and Morphological Characterization of Free MCM-41 and Functionalized Systems 

Morphological characterization was performed using the scanning electron microscope (FE-SEM Sigma VD series, ZEISS, Jena, Germany) operating at 30 kV, as well as a transmission electron microscope (TEM: Tecnai G2-20 SuperTwin, FEI Company, Hillsboro, OR, USA) at 200 kV. The N_2_ adsorption was performed with the equipment Autosorb IQ (Quantachrome Instruments, Boyton Beach, FL, USA), to obtain information on porosity and surface area of MCM-41 before and after addition of APTES and FFA. Samples of MCM-41 were treated at 300 °C for 4 h, whereas functionalized samples received treatment at 40 °C for 48 h. The values of the surface area, pore diameter, and volume were calculated by Brunnauer, Emmett, and Teller (BET) method, except for pore distribution data, which were obtained from Density Functional Theory (DFT) method instead. Small-angle X-ray spectroscopy (SAXS: Ultima IV^®^, Rigaku, Tokyo, Japan) was used to confirm the diffraction pattern of the pores of MCM-41 before and after functionalization with APTES and FFA. The incident X-ray was set at a 1.54 Å wavelength, and the angle scattering (2θ) in the range of 0–6°. Fourier transform infrared spectroscopy (FTIR: Nicolet 6700, Thermo Scientific, Waltham, MA, USA) was performed as a qualitative technique to identify APTES and FFA after the process of functionalization of MCM-41. The samples were analyzed in KBr pellets, with 256 scans at the 4000 to 400 cm^−1^ range at a resolution of 4 cm^−1^. The ratio of MCM-4 to KBr was 1:100 (mg). Thermogravimetric analysis (TGA: DTG-60H, SHIMADZU, Kyoto, Japan) was performed in nitrogen atmosphere at 50 mL/min from 25 to 700 °C at a rate of 10 °C/min. In order to determine the presence of C and N chemical elements, the electron energy loss spectroscopy (STEM-EELS: GIF Quantum SE detector system, Gatan, Pleasanton, CA, USA) was performed with energy resolution of 1.5 eV and dispersion of 0.25 eV/pixel. Energy-filtered transmission electron microscopy (EFTEM) was performed, to increase contrast and retrieve a unique effect that later can be worked upon with ImageJ software, which can assign different colors to chemical elements to allow the merging of the two or more images. Photon correlation spectroscopy and Zeta Potential analysis (Zetasizer Nano ZS, Malvern Instruments, Westborough, MA, USA) allowed to determine the mean diameter and surface charge of nanoparticles. The analytical procedure was conducted after the adequate dilution of the samples in ultra-pure MilliQ^®^ water (0.05 mg/mL). The results were expressed as mean, ± standard deviation, for at least three different batches of each silica nanoparticles formulation. 

### 2.6. Flufenamic Acid Release Test

A suspension of nanoparticles functionalized with FFA (3 mg) in simulated body fluid (SBF) (1 mL, pH 7.35) was put into a dialysis tube (cut-off Mn = 3500-Max Molecular Weight) and then placed in a flask with 24 mL of SBF at 37 °C, under continuous stirring, at a rate of 50 rpm. At particular time intervals, the amount of FFA released from nanoparticles was calculated, by measuring the absorbance at 288 nm with a UV–Vis spectrometer (UV-2550, SHIMADZU, Kyoto, Japan). The in vitro experiments were carried out in triplicate.

### 2.7. Cell Culture 

Human fibroblasts (MRC-5) were cultured in a T75 flask with 10 mL of Dulbecco’s Modified Eagles’s Medium- high glucose (DMEM), to which was added 10% fetal bovine serum and 10 mL of penicillin, streptomycin, and amphotericin (PSA). The cells were maintained in the incubator at 37 °C, 95% humidity, and 5% CO_2_.

### 2.8. Biocompatibility Assay

MRC-5 human fibroblasts cells were seeded at 1.0 × 10^4^ cells per well in a 24-well plate, in the absence or presence of MCM-41, MCM-41/AP, and MCM-41/AP-FFA (10, 50, and 100 µg/mL) for 48 h. Viable and necrotic cells were assessed by calcein and propidium–iodide (PI) staining, respectively. Briefly, 7 µL of calcein AM (1 µM) and 50 µL of PI (1 mg/mL) were added in 7 mL of DMEM. Next, each well received 300 µL of the staining mixture, and was kept in the incubator at 37 °C for 10 min. The images were taken using an inverted fluorescence microscope (Olympus IX70, Olympus, Tokyo, Japan). Calcein AM was detected at 288 nm, and PI at 570 nm. Analysis of the images was performed with ImageJ software. The MRC-5 cells were obtained upon informed consent from the patients, according to procedures approved by Ethics Committee of the Universidade Federal de Minas Gerais (no. ETIC 02887512.6.0000.5149), following the Brazilian laws statements on resolution CNS 196/96 published on 12 September 2011.

## 3. Results and Discussion

### 3.1.Scanning Electron Microscopy (SEM) and Transmition Electron Microscopy (TEM) Results 

**Figure 1:**
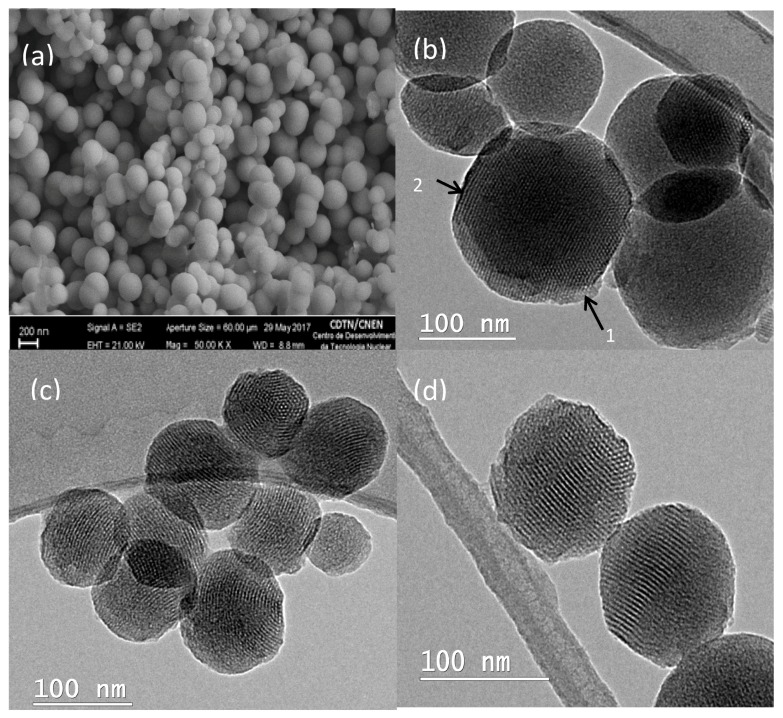
(**a**) Scanning Electron Microscopy (SEM) images of MCM-41 and Transmition Electron Microscopy (TEM) images of (**b**) MCM-41, (**c**) MCM-41/AP, and (**d**) MCM-41/AP–FFA, showing the hexagonal pore arrangement.

The morphological characterization of silica nanoparticles was carried out by SEM and TEM techniques. Images of MCM-41 obtained through SEM showed spherical morphology of most nanoparticles, and low heterogeneity among them (Figure 1a). The particles sizes were measured by Quantikov software (Quantikov Image Analyzer, IPEN, São Paulo, Brazil) [28], presenting an average mean diameter of approximately 140 ± 20 nm. Four images from different regions of the sample support (stub) were loaded into Quantikov software, where a scale was set before measuring. Once the scale was set up, each nanoparticle was marked manually. The program provided the histogram once all the nanoparticules in the images were measured. This size range is desirable in candidate nanomaterials acting in cancer treatment by the EPR effect, because the accumulation of nanoparticles on tumor sites may be favorited by the nanoscale of the porosity of blood vessels that is common on tumors. 

**Figure 2:**
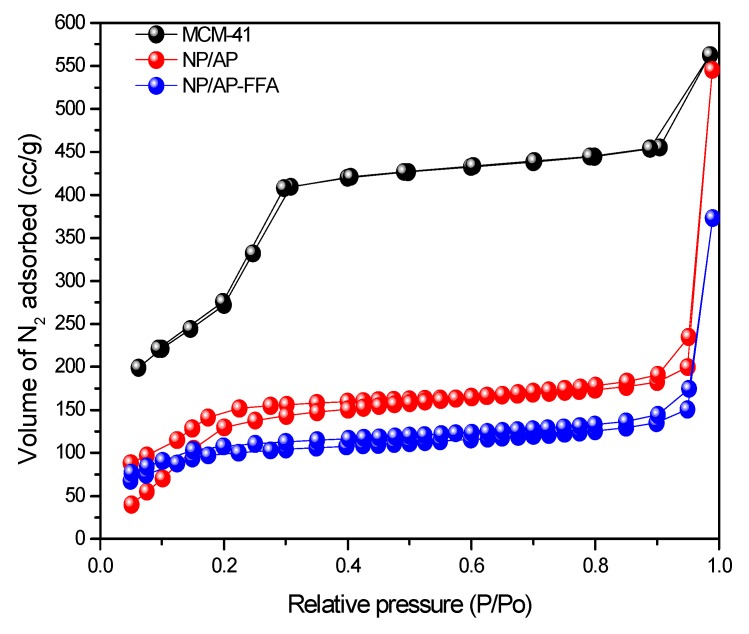
Isotherm of MCM-41, MCM-41/AP and MCM-41/AP-FFA.

The pore arrangement determined by TEM showed a well-defined hexagonal arrangement of uniform pores, observed when the electron beam was parallel to the main axis of the mesopores (Figure 1b-1), and in the (100) direction when the electron beam was perpendicular to the main axis, shown in Figure 1b-2. Figure 1c,d show TEM images of MCM-41/AP and MCM-41/AP–FFA, respectively. Thus, the TEM investigation offers consistent evidence that the ordered structure is preserved in the approach proposed in this work to obtain functionalized nanostructured systems.

### 3.2. Nitrogen Adsorption Analysis

The isotherm obtained from the adsorption of N_2_ technique is characteristic of MCM-41, which matches type IV, and the hysteresis loop obtained from the adsorption fits type H1, according to classification by the International Union of Pure Applied Chemistry (IUPAC). This hysteresis indicates the amphiphilic feature of the mesoporous surface, where there is a capillary condensation of the adsorbate (e.g., N_2_). Firstly, a monolayer forms on the walls of the material, followed by multiple layers that form, as suggested in the results from the graph, where the saturation pressure is reached before the saturation pressure of the gas (Figure 2) [29,30]. Note that the form of the isotherm is not affected by the process of the functionalization of MCM-41/AP and MCM-41/AP-FFA; a reduction of N_2_ adsorption volume for all the relative pressures could be observed, suggesting that the process of chemical surface modification of the nanoparticles had occurred. Such difference might be attributed to the result of FFA chain molecules connected with amino groups, and in the first functionalization process, it could be due to the APTES moieties anchored in the hydroxyl groups. 

Calculations of surface area, pore diameter, and pore volume were determined by the BET method, and Table 1 summarizes these textural properties. The surface area, pore volume, and pore diameter obtained were 1145 m^2^/g, 0.86 cc/g, and 3.2 nm, respectively (Table 1). As expected, the introduction of the organic moieties (APTES and AFF) to the MCM-41 led to a decrease in the surface area and pore volume. After surface amination with APTES, a significant reduction of those parameters to 409 m^2^/g and 0.33 cc/g, respectively, was observed, while the pore diameter remained the same. It is possible that the maintenance of the average diameter is due to the contribution of the average of non-functionalized pores. The functionalization with FFA led to a decrease in the surface area and pore volume to 272 m^2^/g and 0.24 cc/g, respectively, indicating the presence of FFA in the mesopores of MCM-41. This sensible difference observed in the values of surface area and pore volume may be an indication of the presence of a functionalization agent in the pore structure of silica.

### 3.3. Small-Angle X-ray Scattering (SAXS)

**Figure 3:**
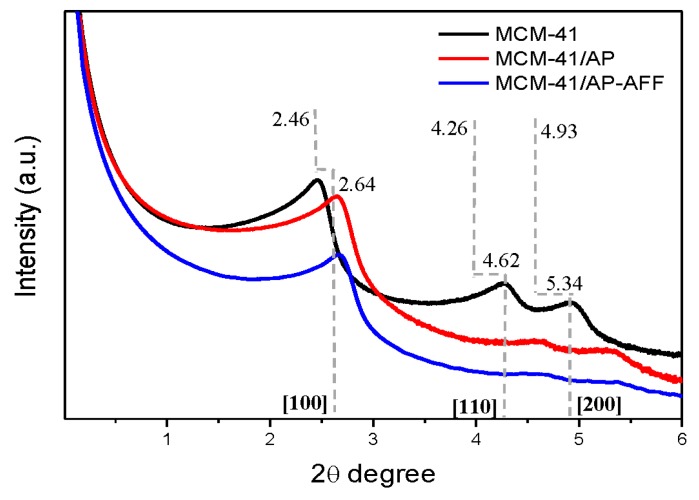
Small angle XRD patterns of MCM-41, MCM-41/AP and MCM-41/AP-FFA.

The pore structure characterization was carried out using small-angle X-ray scattering (SAXS). Figure 3 shows the SAXS patterns of MCM-41 and the functionalized samples. Small-angle XRD of MCM-41 showed a pattern with (100), (110), and (200) reflections at 2θ = 2.46, 4.27, and 4.92, respectively, characteristic of the hexagonal structure of MCM-41 as described in the literature [29]. It is worth nothing that the functionalized samples also exhibited the typical well-ordered hexagonal mesoporous structure, with three distinct diffraction peaks. However, all of the XRD reflection intensities decreased after surface modification, especially for the sample with FFA content. The decrease in peak intensity when additional substances were incorporated within the mesopores, or added to the external surface, could be ascribed to an overall decrease in the electron density contrast between the pore wall and the empty pore [31]. Moreover, shifts of all the reflections could be observed at higher angles, indicating a decrease in mesopore sizes. 

The detailed structural parameters of the materials are given in Table 2. Data showed a decrease in cell unit parameters of functionalized MCM-41, based on the shift of diffraction pattern to higher angles compared to the unfunctionalized samples. The main peak, which corresponds to the 2θ value of the crystallographic plane (100), increased from 2.46 to 2.64, indicating that the initial 2θ is no longer obeying Bragg’s law after functionalization with APTES. A decrease in the interplanar spacing *d* parameter from 3.58 nm to 3.34 nm was expected, since APTES molecules were added in the mesoporous silica nanoparticles surface, including in the mesopores. The cell unit decreased for the (100) plane, from 4.13 nm to 3.85 nm, and the wall thickness *h* from 0.93 nm to 0.85 nm. This may indicate that the layer of APTES deposited on MCM-41 formed another layer that comprised a new and smaller cell unit, resulting in a decrease in pore diameter compared to the MCM-41 sample, according to BHJ calculations (Table 2) and similar results previously reported [17], and in accordance with studies in the literature [32]. Interestingly, grafting with FFA (MCM-41/AP-AFF) did not further change the parameters of MCM-41 compared to its MCM-41/AP counterpart, indicating that the presence of FFA did not affect the cell unit parameters and only decreased the pore volume, whereas the diameter remained 3.0 nm. 

### 3.4. Fourier Transform Infrared Spectroscopy (FTIR)

**Figure 4:**
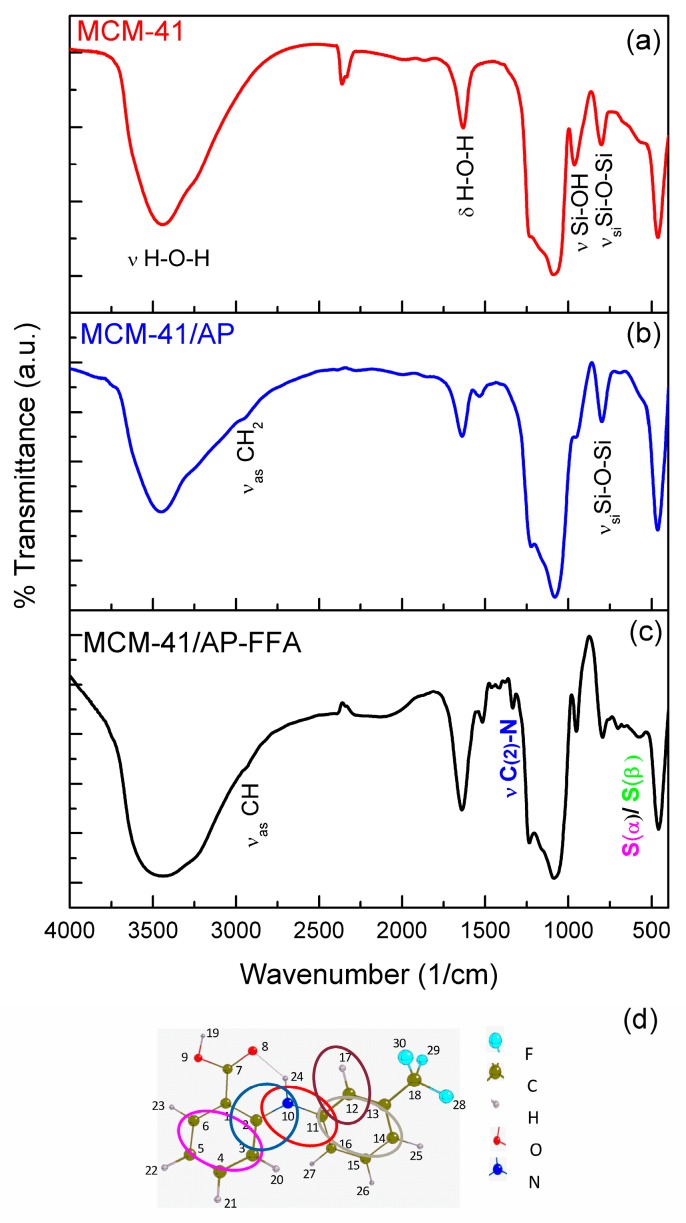
Fourier transform infrared spectroscopy (FTIR) survey spectra of (**a**) MCM-41, (**b**) MCM-41/AP, (**c**) MCM-41/AP-FFA, and (**d**) Molecular structure of FFA.

FTIR spectra of was used to identify functional groups in MCM-41 nanoparticles, as well as their functionalized counterparts. The main peaks of MCM-41 were detected at 799 cm^−1^, assigned to symmetric stretching (ν_s_) Si-O-Si and its bending (δ) at 465 cm^−1^ [31]. A wide peak between 3000 cm^−1^ and 3600 cm^−1^ refers to the stretching of (ν) H-O-H, due to the water adsorbed onto the surface of MCM-41 (Figure 4a). 

In addition, after functionalization with APTES (MCM-41/AP), there is a modification in the shape of 950 cm^−1^ peak (Si-OH), showing less definition than in the MCM-41 spectrum, indicating a decrease on silanol groups, as expected after the amination process [33]. The consumption of silanol groups during the interaction of the alkoxysilanes from APTES with the surface of the MCM-41 was expected, and it is possible to observe the formation of a new band at 1532 cm^−1^, indicating the presence of a C-N bond at the aminated sample. This indicates that the adsorption of organic groups on the silica surface happens simultaneously with the disappearing of hydroxyl groups.

In Figure 4c, it is possible to observe the formation of two more bands at 1339 cm^−1^ and 576 cm^−1^, indicating the presence of C(2)-N and benzene rings S(α)/S(β) characteristic of an FFA molecule, suggesting that the functionalization process was successful.

**Figure 5:**
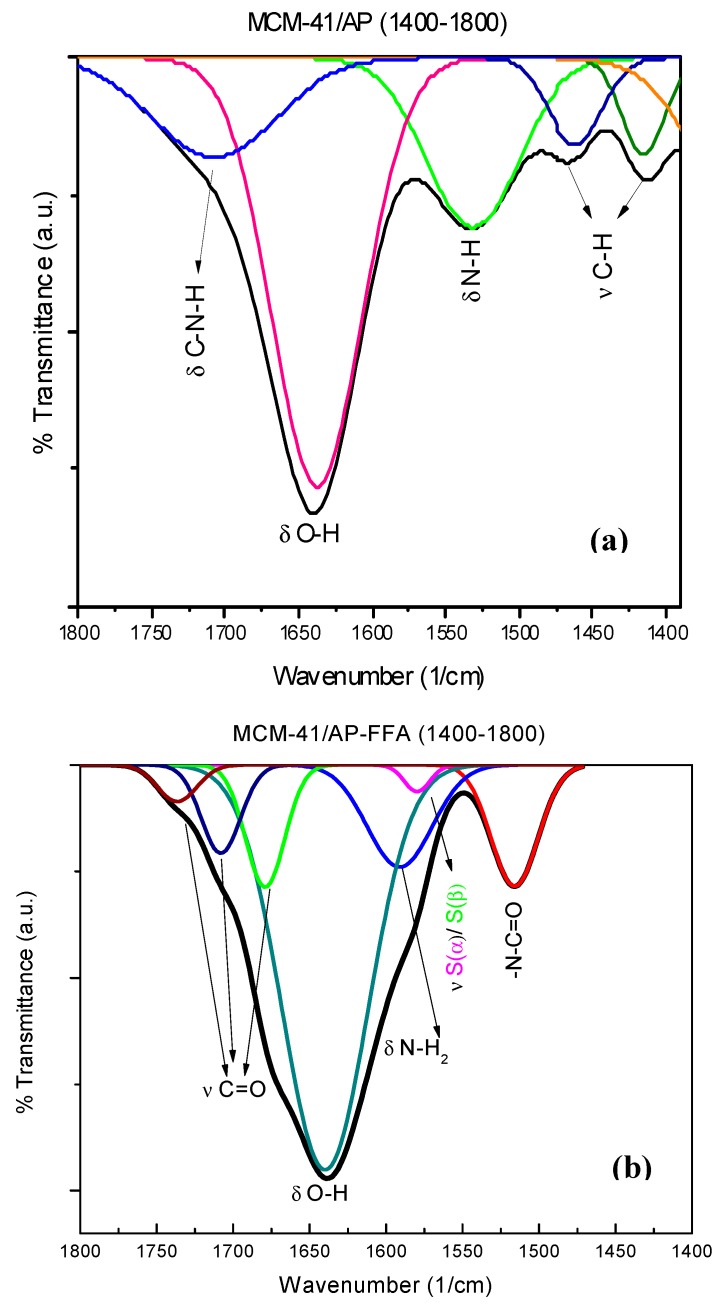
Refined FTIR spectra of the 1400–1800 cm^−1^ bracket. (**a**) MCM-41/AP; (**b**) MCM-41/AP-FFA.

When region I (1400 cm^−1^–1800 cm^−1^) was subjected to refinement through PeakFit v4 (PeakFit v4, Systat, San Jose, CA, USA) a change in band pattern could be observed between the samples of MCM-41/AP and MCM-41/AP-FFA. In the former sample, four peaks appeared at 1708 cm^−1^, 1639 cm^−1^, 1531 cm^−1^, 1465 cm^−1^, and 1412 cm^−1^, assigned to the bending of C-N-H bound, O-H vibration, N-H bending, and C-H stretching, respectively (Figure 5a), confirming the presence of APTES [34,35,36]. The last two groups were present in the APTES, confirming that the surface of the MCM-41 was aminated. 

In the refined spectra, obtained after further grafting with FFA, three peaks at 1679 cm^−1^, 1708 cm^−1^, and 1736 cm^−1^ could be observed, which are all related to the stretching of the (ν)C=O of FFA molecule (Figure 5b). Moreover, peaks at 1591 cm^−1^, 1579 cm^−1^, and 1515 cm^−1^, assigned to (δ)N-H_2_, S(α)/S(β), and N-C=O can be seen. The (δ)N-H_2_ is likely to be the secondary amine of FFA. S(α)/ S(β) and N-C=O peaks are another detectable vibration wavelength for the benzene rings of FFA, and there may be an amide group as a result of the reaction between carboxyl from FFA and amine groups from APTES. The presence of all the peaks described above supports the success of the grafting process in MCM-41 [37].

### 3.5. Electron Energy Loss Spectroscopy (EELS) and Energy-Filtered Transmission Electron Microscopy (EFTEM)

**Figure 6:**
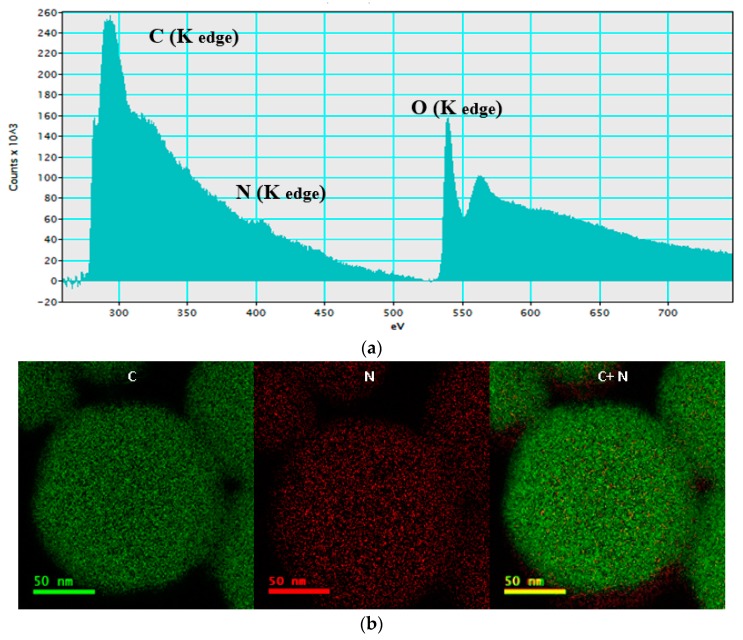
(**a**) Electron energy loss spectroscopy (EELS) spectra of functionalized with flufenamic acid (FFA) (MCM-41/AP-FFA), and (**b**) energy-filtered transmission electron Mmcroscopy (EFTEM) images obtained by TEM.

Analysis of the MCM-41/AP-FFA sample by electron energy loss spectroscopy (EELS) showed a strong and sharp peak assigned to carbon (C) at 284 eV (K edge), a second weak peak at 401 eV, which corresponds to nitrogen (N), and a third main sharp peak at 540 eV (Figure 6a). C and N elements detected were present in the APTES and FFA molecules, thus confirming the organic component of the samples. The oxygen (O) peak is part of the silica dioxide (SiO_2_) structure of the MCM-41. Based on the data gathered from EELS, it was possible to determine the distribution of C and N in the MSNs. A homogeneous distribution of both elements can be seen in Figure 6b. As expected, more C were seen (in green) than N (in red), showing that the proportion of C to N in APTES and FFA molecules was high.

### 3.6. Thermogravimetric Analysis (TGA-DTG)

**Figure 7:**
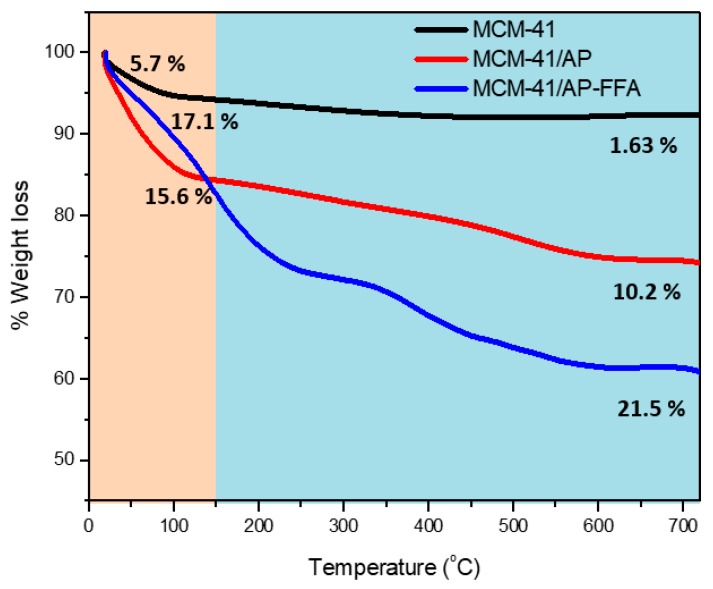
Thermogravimetric analysis (TGA) graph of mass loss (%) of MCM-41, MCM-41/AP, and MCM-41/AP-FFA.

Thermogravimetric analysis (TGA) was carried out to determine the presence of organic compounds in the MCM-41, before and after chemical modification steps with APTES and FFA. The TGA graph shows the profile of mass loss curves, where the lines representing each sample register the increase in mass loss from MCM-41 towards the grafted samples MCM-41/AP and MCM-41/AP/FFA (Figure 7). Two temperature regions were chosen. The region between 25 and 150 °C represented the region of water loss (in light pink), while the region from 150 °C on (in blue) represented the region of loss of the organic components grafted to the MCM-41 nanoparticles. The MCM-41 showed a 5.7% loss of adsorbed water from 25 to 150 °C (Table 3). The functionalized samples MCM-41/AP and MCM-41/AP-AFF lost more water. From 150 to 700 °C, the MCM-41 sample lost only approximately 1.63% of mass, probably related to traces of CTAB used during the synthesis of the silica nanoparticles. 

After functionalization with APTES, the mass loss between 150 and 700 °C increased to 10%, confirming the presence of APTES in the MCM-41. The MCM-41/AP-FFA sample lost 21% of its mass within the same temperature bracket. This result confirms the presence of FFA, counting for 10% variation (Δ) compared to MCM-41/AP [18,34].

**Figure 8:**
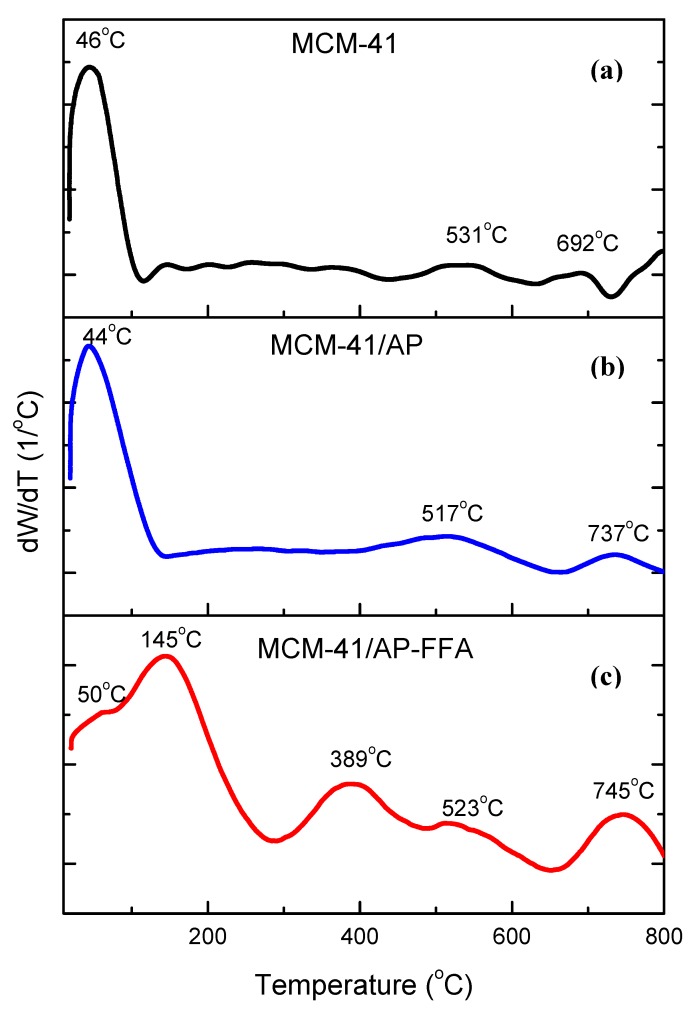
DTG of (**a**) MCM-41, (**b**) MCM-41/AP, and (**c**) MCM-41/AP-FFA.

The Derivative Thermogravimetric Analysis (DTG) provides the temperature brackets where mass loss occurred. All the analyzed samples presented a mass loss within 45.4 °C and 52.6 °C, related to the water adsorbed in the surface of nanoparticles (NPs) (Figure 8). Above 650 °C, organic components start decomposing. The presence of two weak and wide peaks at 531 °C and 692 °C in MCM-41 may be due to the traces of CTAB (Figure 8a). A peak at 517 °C in the MCM-41/AP indicates the decomposition of APTES, and another peak at 737 °C (Figure 8b), likely to be APTES, reacted more strongly to NP. Further functionalization with FFA showed a peak at 145 °C, suggesting the loss of FFA that is interacting more loosely to nanoparticles (Figure 8c). Moreover, there was another peak at 389 °C, which may be the FFA strongly interacting with the molecular structure of NPs, while the other two peaks at 523 °C and 745 °C represent the loss of APTES. The presence of more than one peak above 600 °C in all samples with APTES may be an indicator that its decomposition may occur in more than one step [23,38].

Additionally, in a previous work, the present study’s authors have reported the loss of APTES by TGA in an N_2_ atmosphere [38], and another group has showed that there is little change in the TG profile of grafted silica nanoparticles done under O_2_, although the final mass loss content did not present a significant difference [39]. 

### 3.7. Elemental Analysis 

Elemental analysis was carried out, in order to determine the presence of APTES and FFA in the grafted samples, and the data are presented in detail in Table 4. The MCM-41 showed only traces of C and H. The carbon is likely due to remnants of CTAB, while the hydrogen maybe from silanol groups, although both detections are below the margin of error of the equipment. After functionalization with APTES, the content of the C element appeared to be 4.7%, while H was 2.7%, and N was 1.5%, which indicates that the APTES was present. Further grafting with FFA led to an increase in the C content to 12.5%, and a discrete increase in the N content to 1.6%. This result indicates the presence of FFA, since it contains C in its benzene rings and N in the secondary amine, between the benzene rings. The total amount of FFA grafted to the MCM-41 was 131 mg/g of the nanoparticles. These results corroborate with mass loss seen in TGA. 

### 3.8. FFA Release Assay

**Figure 9:**
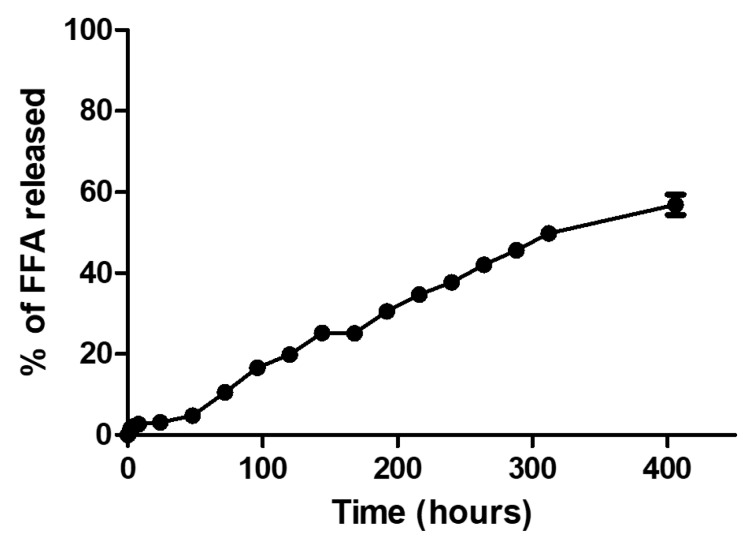
Release profiles of FFA from MCM-41.

The in vitro FFA release behavior was investigated as a function of time, and the result is shown in Figure 9. The release of FFA was very slow, as indicated by analyzing the results, and a very small proportion of FFA was released. It is worth noting that first there was a plateau, and later the release started increasing again. It might be possible that this first release was FFA trapped or covalently bound to the APTES, which formed smaller cell units as indicated by SAXS results, and the later increase was the FFA being released from the bigger cell units. A second possible explanation for this slow release may have been the pH value. Finally, it is possible that the FFA could have been easily hydrolyzed in the buffer used for the assay, which would have compromised the detection of the intact molecule at 288 nm [40,41]. However, the antioxidant role of FFA may be carried out when still interacting or trapped inside the MCM-41, within the 48 h chosen as the timepoint for the in vitro biocompatibility assay, as shown below. The same behavior is possible in vivo, once the timepoint is long enough to allow for the endocytosis of the MSN by the cells, through mechanisms yet to be elucidated, where the FFA is likely to scavenge the free radicals, in case the cells are exposed to harmful stimuli that may tip the oxidation/reduction balance, leading to apoptosis of normal cells [42]. Moreover, a similar trend in drug release was observed for another non-steroidal drug analog to FFA—mefenamic acid—showing a first-order release profile [43].

### 3.9. Biocompatibilidade Assay

**Figure 10:**
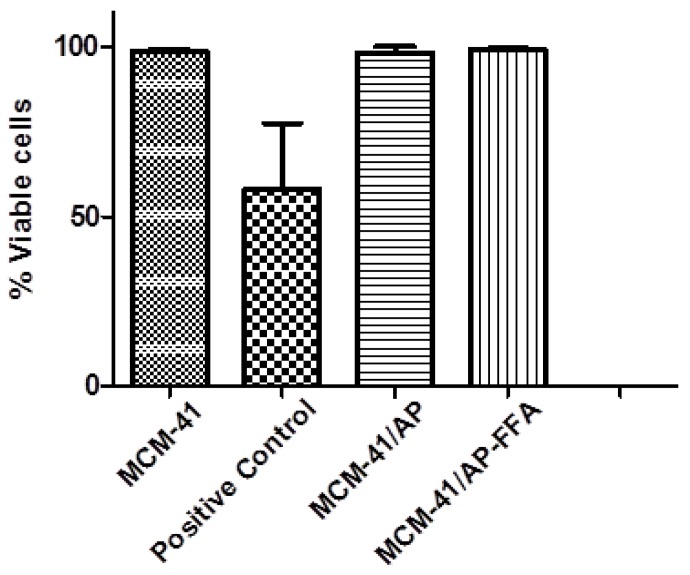
Biocompatibility graph for MRC-5 cells treated with MCM-41, MCM-41/AP, and MCM-41/AP-FFA.

In order to access the biocompatibility of MCM-41 and its functionalized counterparts, human fibroblasts MRC-5 cells were cultured in the presence of each material (MCM-41, MCM-41/AP, and MCM-41/AP-AFF) at 50 µg/mL for 48 h. Later, each group was stained with Calcein and PI to indicate live and dead cells, followed by fluorescence imaging. All treated groups showed a high biocompatibility compared to the control groups. Cell viability of MRC-5, in the presence of MCM-41, MCM-41/AP, and MCM-41/AP-AFF remained between 95% and 99% compared to control groups (Figure 10). This is advantageous, given that after the release of FFA, the remaining aminated MCM-41 is unlikely to cause damage to the cells, and counteract the positive effect of FFA. Neither the presence of MCM-41, nor MCM-41/AP, nor MCM-41/AP-FFA was toxic to the MRC-5 cells, as seen in images where the cells remained strongly stained with calcein (green), and nearly no detectable cells stained for PI (red). According to Zhimin Tao and coworkers [44], the positively-charged groups on the mesoporous nanoparticles could be inclined to bind to the negatively-charged cell membrane, instead of bringing the materials into the cytoplasm. This actual failure in endocytosis may protect the cells from serious injury. 

**Figure 11:**
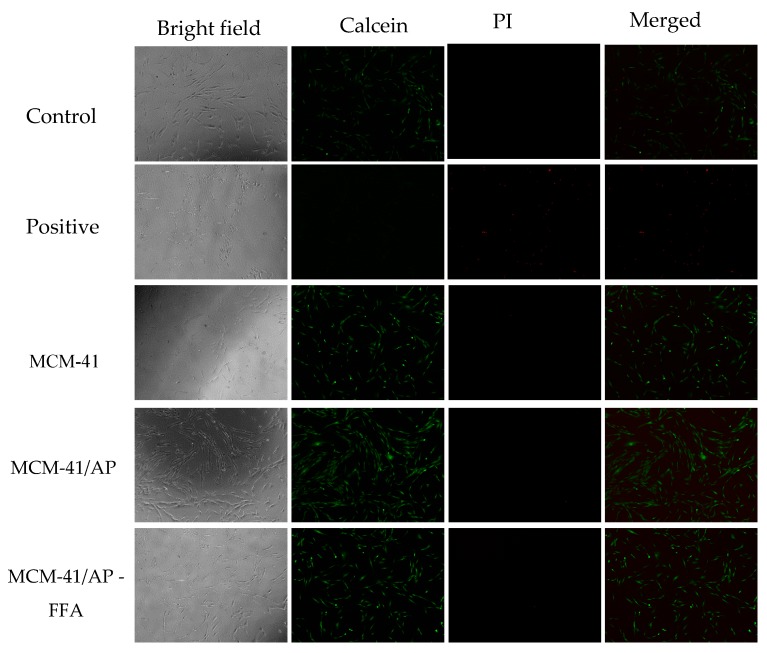
Images from fluorescence microscopy for all groups. Calcein (green) for viable (alive) cells, and propidium–iodide (PI) (red) for dead cell staining.

Moreover, the contact of the cells with MCM-41, or MCM-41/AP and MCM-41/AP-AFF, did not lead to morphological changes, as seen in the bright-field images obtained by fluorescence microscopy. The positive control group was the only one that showed morphological changes, due to the treatment with cisplatin (5 µg/mL), where a large number of dead cells stained with PI could be seen. Results are shown in Figure 11. These results are important with respect to the purpose of this study aiming at protecting normal cells. The nanoparticles carrying the FFA are supposed to accumulate around the tumor, due to the EPR effect, and from there release the drug to protect the normal cells from damaging signaling molecules, released by cancerous cells to genomically destabilize the normal cells. Although the MCM-41/AP nanoparticles are positively charged, according to zeta potential measurements, there is a decreasing in this value after grafting with FFA (see Table 5). Their hydrodynamic size increased after addition of APTES, to 242 nm, and decreased slightly to 210.2 nm in the presence of FFA. This indicates that the nanoparticles maintained their stability after the functionalization process. Our group has reported a good biocompatibility of our silica nanoparticles. Besides, it is worth noting that there is a possibility of the formation of a protein corona, since the samples were dispersed in serum-enriched DMEM. It common in the literature that the corona formed around the nanoparticles lowers its cytotoxicity [43,45,46,47] by lowering its positively-charged surface. 

## 4. Conclusions

In closing, the characterization techniques showed that functionalization of MCM-41 with FFA is possible, and still allows for tailoring as to the amount of APTES and FFA that can be grafted onto the surface of MCM-41. The treatment of MRC-5 cells with MCM-41, MCM-41/AP, and MCM-41/AP-FFA showed high biocompatibility (up to 99%), to the point where necrotic cells could hardly be seen. Such behavior is essential for the potential use of silica mesoporous grafted with FFA for the protection of normal cells from damaging molecules released by cancer cells. The EPR effect would lead to accumulation of those silica nanoparticles around the tumor, where it indicates a beneficial effect on normal cells while increasing cell death on cancerous ones.

## Figures and Tables

**Table 1:** Data of surface area, pore diameter, and pore volume, before and after functionalization of MCM-41 with 3-Aminopropyltriethoxysilane (APTES) and flufenamic acid (FFA) from N_2_ adsorption results.

Sample	Surface Area (m^2^/g)	Pore Diameter (nm)	Pore Volume (cc/g)
MCM-41	1145	3.2	0.87
MCM-41/AP	409	3.0	0.33
MCM-41/AP–FFA	272	3.0	0.24

**Table 2:** Structural properties of the MCM-41 and functionalized samples calculated, from the positions of the (110) plane reflections.

Sample	2θ_d100_	d_100_ (nm)	a_100_ (nm)	h (nm)
MCM-41	2.46	3.58	4.13	0.93
MCM-41/AP	2.64	3.34	3.85	0.85
MCM-41/AP-FFA	2.64	3.34	3.85	0.85

**Table 3:** Weight loss of MCM-41, MCM-41/AP, and MCM-41/AP-FFA.

Sample	25–150 °C	150–700 °C
MCM-41	5.7%	1.63%
MCM-41/AP	15.6%	10.0%
MCM-41/AP-FFA	17.1%	21.5%

**Table 4:** Percentages of chemical elements carbon, hydrogen, and nitrogen in MCM-41, MCM-41/AP, and MCM-41/AP-FFA, given by elemental analysis.

Sample	Carbon (C)			Hydrogen (H)			Nitrogen (N)		
	%	mmol·g^−1^	∆ C	%	mmol·g^−1^	∆ H	%	mmol·g^−1^	∆ N
MCM-41	0.1			0.2					
MCM-41/AP	4.7	3.9	4.6	2.7	27.0	2.5	1.5	1.0	1.6
MCM-41/AP-AFF	12.5	10.4	7.9	2.5	25.0		1.6	1.1	0.1

**Table 5:** Zeta potential and hydrodynamic size of MCM-41, MCM-41/AP, and MCM-41/AP-AFF.

Sample	Zeta Potential	Hydrodynamic	Polydispersion Index (PDI)
MCM-41	−23.2 meV	130 nm	0.4
MCM-41/AP	+39 meV	242 nm	0.2
MCM-41/AP-AFF	+36 meV	210 nm	0.2
